# Supplementary figures and images for: Economic evaluation of participatory women’s groups scaled up by the public health system to improve birth outcomes in Jharkhand, eastern India
Source: PLOS Glob Public Health. 2023 Jun 29;3(6):e0001128. doi: 10.1371/journal.pgph.0001128 (PMC10309599; doi:10.1371/journal.pgph.0001128)

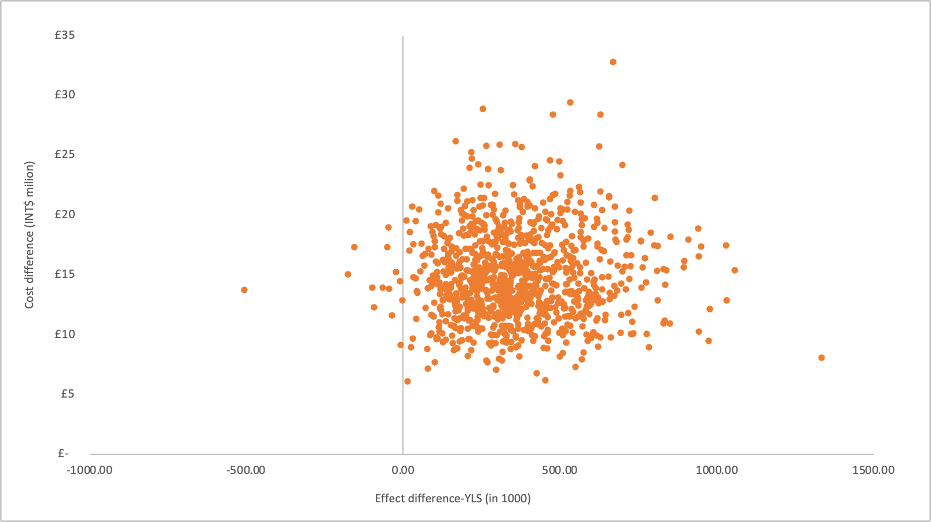

Supplement: S1 Fig — (TIFF) [file pgph.0001128.s002.tiff]

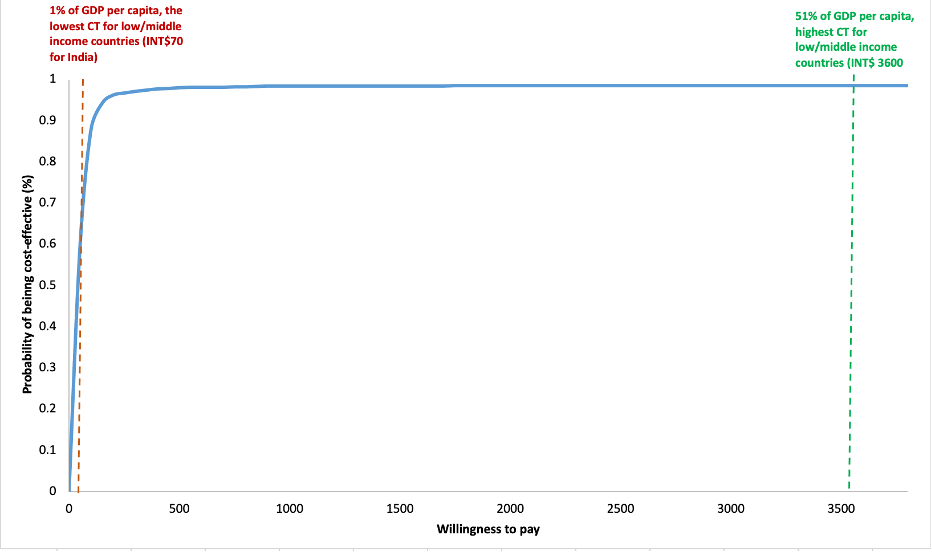

Supplement: S2 Fig — (TIFF) [file pgph.0001128.s003.tiff]

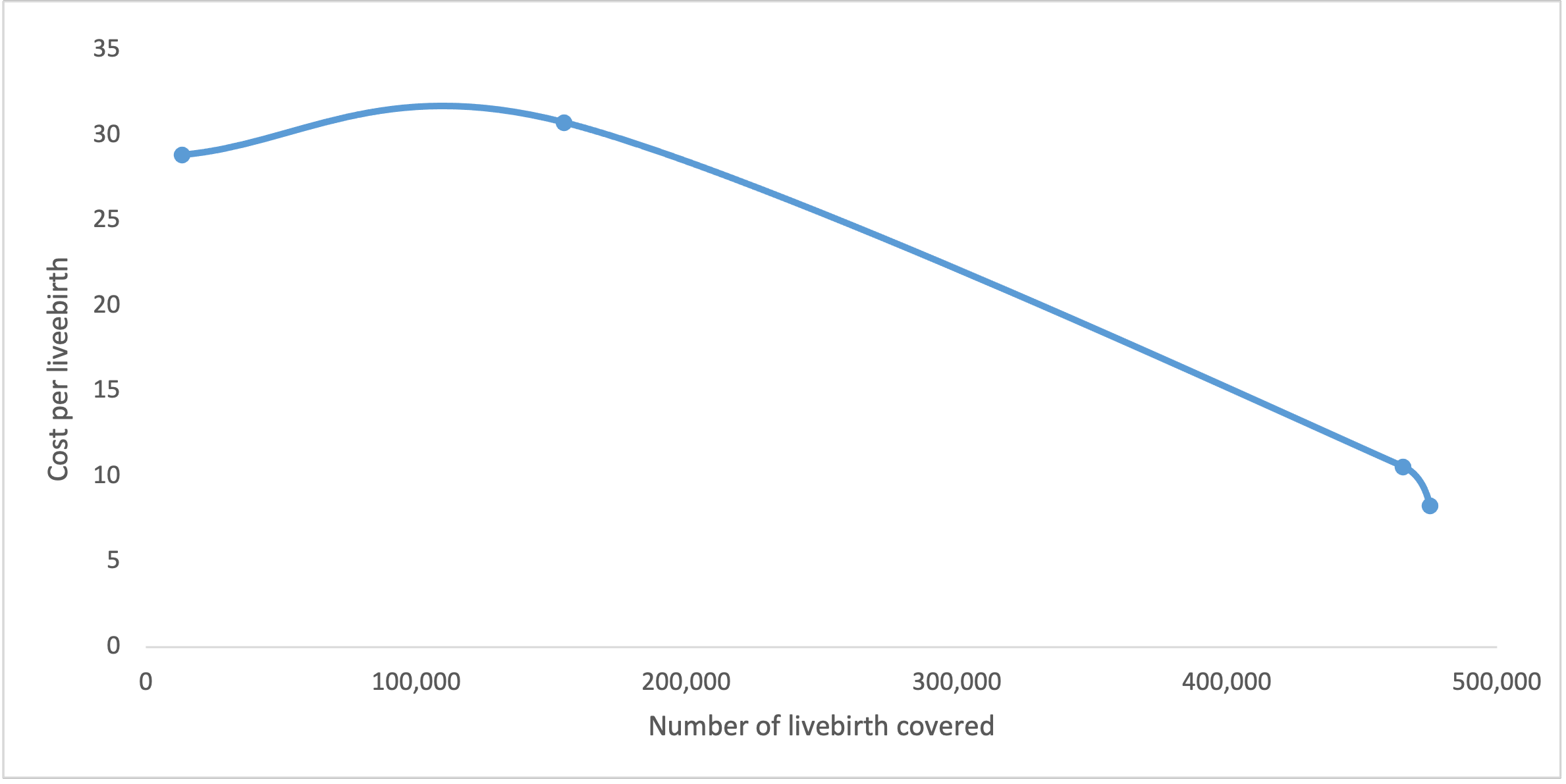

Supplement: S3 Fig — (TIFF) [file pgph.0001128.s004.tiff]
