# Supplementary material for: Economic evaluation of participatory women’s groups scaled up by the public health system to improve birth outcomes in Jharkhand, eastern India
Source: PLOS Glob Public Health. 2023 Jun 29;3(6):e0001128. doi: 10.1371/journal.pgph.0001128 (PMC10309599; doi:10.1371/journal.pgph.0001128)
Supplement: S1 Table — (DOCX) [file pgph.0001128.s005.docx]

S1 Table: Parameters used in deterministic and probabilistic sensitivity analyses of FLAG

| Parameter | Values | | | Distribution for PSA | Source | Results for one-way sensitivity analysis (INT$) | |
| --- | --- | --- | --- | --- | --- | --- | --- |
|  | Base-case | Low | High |  |  | Low | High |
| Intervention effectiveness (risk ratio) | 0.77 | 0.60 | 0.98 | Lognormal | Point estimate and 95% CI from the FLAG impact evaluation(1) | 24 | 475 |
| Intervention costs (+/-25%) | 15,017,396 | 11,263,047 | 18,771,745 | Lognormal | Assumption | 31 | 52 |
| Crude Birth Rate in rural area | 0.02 | 0.014 | 0.027 | Lognormal | 0.014 and 0.027 are the lowest and the highest crude birth rate in rural India in 2018(2). | 31 | 59 |
| NMR in rural areas with no intervention | 0.032 | 0.0183 | 0.0523 | Lognormal | NMR of 0.032 was average NMR over 2016-2020 period.  0.0183 and 0.0523 were the lowest and the highest NMR for 24 districts of Jharkhand between 2005-2015, presented in Nair et al(1), Supplementary Table 5 (Data from NFHS-4. As reported in Bora & Saikia (2018)(3)- ) | 25 | 73 |
| Life expectancy at birth | 86 | 70 | 91.9 | N/A | 86: Standard life expectancy recommended by GBD2010(4); 70: 2019 India life expectancy at birth(5); 91.9: WHO Global Health Estimates(6) | 41 | 44 |
| Discount rate | Costs 3%, life-years 3% | Costs 0%, life-years 0% | Costs 10%, life-years 3% | N/A | Based on recommendation of iDSI reference case(7) and Haacker et al(8) | 16 | 36 |

**References:**

1.Nair N, Tripathy PK, Gope R, Rath S, Pradhan H, Rath S, et al. Effectiveness of participatory women's groups scaled up by the public health system to improve birth outcomes in Jharkhand, eastern India: a pragmatic cluster non-randomised controlled trial. BMJ Glob Health. 2021;6(11).

2.Office of the Registrar General & Census Commissioner. SRS Statistical Report 2018: ESTIMATES OF FERTILITY INDICATORS 2018.

3.Bora JK, Saikia N. Neonatal and under-five mortality rate in Indian districts with reference to Sustainable Development Goal 3: An analysis of the National Family Health Survey of India (NFHS), 2015-2016. PLoS One. 2018;13(7):e0201125.

4.Murray CJL, Ezzati M, Flaxman AD, Lim S, Lozano R, Michaud C, et al. GBD 2010: design, definitions, and metrics. The Lancet. 2012;380(9859):2063-6.

5.Life expectancy at birth, total (years) - India [Internet]. 2020 [cited 10/11/2020]. Available from: <https://data.worldbank.org/indicator/SP.DYN.LE00.IN?locations=IN>.

6.World Health Organization. WHO methods and data sources for global burden of disease estimates 2000-2015. Global Health Estimates Technical Paper. Geneva: Department of Information, Evidence and Research, WHO; 2017.

7.Wilkinson T, Sculpher MJ, Claxton K, Revill P, Briggs A, Cairns JA, et al. The International Decision Support Initiative Reference Case for Economic Evaluation: An Aid to Thought. Value Health. 2016;19(8):921-8.

8.Haacker M, Hallett TB, Atun R. On discount rates for economic evaluations in global health. Health Policy Plan. 2020;35(1):107-14
