# Supplementary material for: Economic evaluation of participatory women’s groups scaled up by the public health system to improve birth outcomes in Jharkhand, eastern India
Source: PLOS Glob Public Health. 2023 Jun 29;3(6):e0001128. doi: 10.1371/journal.pgph.0001128 (PMC10309599; doi:10.1371/journal.pgph.0001128)
Supplement: S2 Table — (DOCX) [file pgph.0001128.s006.docx]

S2 Table: Cost and cost-effectiveness results of FLAG (PLA at scale) vs. small scale efficacy PLA trials

|  | FLAG | India (Tripathy, et al, 2010) | Nepal (Manandhar et al, 2004) | Malawi (Lewycka et al, 2013) | Malawi (Colbourn et al, 2013) | Bangladesh I (Azad et al, 2010) | Bangladesh II (Fottrell et al, 2013) |
| --- | --- | --- | --- | --- | --- | --- | --- |
| Number of beneficiaries | | | | | | | |
| Total population covered | 24,260,836 | 114,141 | 86,704 | 94,992 | 1,200,000 | 229,195 | 243,341 |
| Total livebirth covered | 1,593,348 | 9,469 | 2,899 | 9,174 | 100,000 | 15,153 | 8,819 |
| Cost per beneficiary | | | | | | | |
| Average annual cost | 4,433,165 | 208,647 | 416,095 | 211,307 | 799,617 | 270,536 | 658,764 |
| Cost per livebirth | 9.4 | 84 | 537 | 193 | 61 | 92 | 254 |
| Cost per person (all ages) | 0.6 | 7.0 | 17.9 | 19.0 | 5.1 | 6.1 | 9.2 |
| Average annual cost per person | 0.2 | 1.7 | 4.5 | 4.2 | 1.3 | 1.1 | 2.6 |
| Average annual cost per livebirth | 2.8 | 20.6 | 134.2 | 42.2 | 15.3 | 16.7 | 72.5 |
| Cost effectiveness ratios | | | | | | | |
| Cost per neonatal death averted | 1,272 | 4,173 | 50,126 | 23,666 | 8,768 | 24,255 | 19,519 |
| Cost per neonatal life year saved | 41 | 135 | 1,627 | 768 | 285 | 787 | 634 |
